# ATF4 Contributes to Ovulation via Regulating COX2/PGE2 Expression: A Potential Role of ATF4 in PCOS

**DOI:** 10.3389/fendo.2018.00669

**Published:** 2018-11-15

**Authors:** Fangfang Di, Jiansheng Liu, Shang Li, Guangxin Yao, Yan Hong, Zi-Jiang Chen, Weiping Li, Yanzhi Du

**Affiliations:** ^1^Center for Reproductive Medicine, School of Medicine, Ren Ji Hospital, Shanghai Jiao Tong University, Shanghai, China; ^2^Shanghai Key Laboratory for Assisted Reproduction and Reproductive Genetics, Shanghai, China; ^3^The Key Laboratory for Reproductive Endocrinology of Ministry of Education, Shandong Provincial Key Laboratory of Reproductive Medicine, National Research Center for Assisted Reproductive Technology and Reproductive Genetics, Center for Reproductive Medicine, Shandong Provincial Hospital, Shandong University, Jinan, China

**Keywords:** ATF4, ovulation, human granulosa cells, COX2, polycystic ovary syndrome

## Abstract

Ovulatory disorder is common in patients with hyperprolactinemia or polycystic ovary syndrome (PCOS). Previous studies have shown that ATF4 plays critical role in apoptosis and glucose homeostasis, but its role in regulating reproductive function was not explored. The present study investigated the role of ATF4 in ovarian ovulatory function. Human granulosa cells (hGCs) from 48 women newly diagnosed with PCOS and 37 controls were used to determine ATF4 expression. *In vitro* cultured hGCs were used to detect the upstream and downstream genes of ATF4. A shRNA- *Atf4* lentiviral vector (sh*Atf4*) was injected into rat ovaries to establish an *in vivo* gene knockdown model to further assess the *in vivo* relevance of the results from PCOS women. We found that ATF4 expression was lower in hGCs from PCOS patients than in hGCs from non-PCOS women. Many pivotal transcripts involved in cumulus-oocyte complex (COC) expansion, extracellular matrix (ECM) remodeling, and progesterone production were significantly down-regulated after *ATF4* knockdown. ChIP-qPCR assays indicated that ATF4 could directly bind to the *COX2* promoter and that *ATF4* knockdown could attenuate human chorionic gonadotropin (hCG)-induced COX2 expression and PGE2 production. The *in vivo* study showed that shRNA-lentivirus mediated *Atf4* knockdown in rat ovaries led to reduced number of retrieved oocytes. Collectively, these findings suggested previously unknown roles of ATF4 in ovulation. Furthermore, ATF4 malfunction in PCOS patients may impact the ovulation process, which could contribute, in part, to the pathogenesis of PCOS.

## PrÉcis

ATF4 in hGCs of PCOS patients was decreased, which impeded hCG-induced COX2 expression and PGE2 production by reducing transcriptional activation and reduced the number of retrieved oocytes in rats.

## Introduction

PCOS, also called Stein-Leventhal syndrome, is an endocrine disorder characterized by oligo-ovulation or continuous anovulation, insulin resistance, and hyperandrogenism. PCOS is common in adolescent and reproductive women and has a morbidity of 9–18% (1, 2). Previous studies have verified that multiple genes correlated with hormone biosynthesis and ovulation are significantly altered in hGCs (3, 4). For the release and transport of oocytes through the oviduct in fertilization, it is essential that the COC matrix is well composed and organized with proper expansion (5). Diminished COC expansion may partially explain the anovulation in women with PCOS (6). However, the molecular mechanism of reduced ovulation in PCOS remains unclear.

Activating transcription factor 4 (ATF4), also known as CREB2, is constitutively expressed in a wide variety of tissues (7). Accumulating evidence suggests that ATF4 plays an important role in regulating the high-level proliferation that is required during osteoblast differentiation (8). ATF4 is able to form heterodimers with members of C/EBP families, including C/EBPα and C/EBPβ, which are essential for ovulation (9, 10). Several studies also identified the role of ATF4 in reproduction. Decreased ATF4 expression in chorionic villus tissue could trigger miscarriage in pregnant women (11). Recently published studies revealed the role of ATF4 in regulating follicular function. For instance, altered expression of ATF4 closely correlated with the development of follicles (12–14). Besides, ATF4 could be induced in the functional and early regression stages of the corpus luteum (CL) (15). However, the specific regulatory mechanisms underlying the function of ATF4 in ovulation remain unclear. The abnormal gene expression profiles of the granulosa cells and ovaries from PCOS patients have revealed many differentially expressed genes (4, 16, 17). Utilizing bioinformatics methods (DAVID Bioinformatics Resources 6.7), we screened for transcription factors that can trigger differentially expressed genes (data not shown). Among those transcript factors, ATF4 was singled-out due to high frequency of its occurrence in the gene expression profiles. Therefore, based on the published microarray data, we hypothesized that ATF4 may play a critical role in PCOS patients.

Cyclooxygenase-2 (COX2) is the key enzyme responsible for the conversion of arachidonic acid into prostaglandins, among which prostaglandin E2 (PGE2) plays important role in multiple physiological and pathological events. hCG can induce COX2 expression and PGE2 production (18, 19). It was reported that COX2-deficient mice were susceptible to reproductive malfunction, such as ovulation disorder, decreased fertilization rate, and embryo implantation dysfunction. Thus, COX2 and PGE2 are markers for evaluating ovulation function (20). A previous study revealed that ATF4 could up-regulate COX2 expression in the kidneys (21, 22). These findings emphasized a close correlation between ATF4 and COX2. ATF4 is also known to participate in HBx-mediated COX2 induction via binding to the *COX2* promoter (23). These findings strongly indicated that ATF4, the upstream regulator of COX2, may play a pivotal role in fertilization.

## Materials and methods

### Recruitment of patients

Eighty five female participants were randomly recruited from the Center for Reproductive Medicine, Ren Ji Hospital, School of Medicine, Shanghai Jiao Tong University, between September 2016 and September 2017. Forty eight of the participants, who were between 20 and 35 years old, were diagnosed with PCOS according to the Rotterdam criteria (oligo- and/or anovulation; clinical and/or biochemical signs of hyperandrogenism; and polycystic ovaries with the exclusion of other causes of hyperandrogenism, such as hyperprolactinemia, androgen-secreting tumors, Cushing's syndrome, and non-classical congenital adrenal hyperplasia) and received *in vitro* fertilization-embryo transfer (IVF-ET) (24). The diagnosis of PCOS was satisfied when two or more of the three criteria were met. The remaining 37 participants in the non-PCOS group were healthy females with regular menstrual cycles (26–35 days) and normal ovarian morphology, and were recruited during visits for routine physical examination, tubal factor infertility, or husband's infertility. Endocrine parameters were measured for the non-PCOS women to exclude hyperandrogenism. None of the participants had received hormonal therapy for at least 3 months before the study. All subjects were of Han ethnicity and underwent gonadotrophin-releasing hormone agonist (GnRHa) protocols. After adequate follicle development, hCG (Lvzhu, China) was administered to trigger ovulation. Oocyte retrieval was performed at 36 h after hCG administration. The basal serum hormonal profiles including FSH, LH, Testosterone (T), estradiol (E2) and prolactin (PRL) were determined using chemiluminescence assay kits (Roche Diagnosis Mannheim, Germany) on the Cobas 6000 analyzer (Roche). Anti-Mullerian hormone (AMH) assay was detected using an ultrasensitive two-site ELISA (AnshLabs, USA) following the manufacturer's protocol. USG was done for the antral follicle count (AFC). The study was approved by the ART Ethics Committee of Ren Ji Hospital, School of Medicine, Shanghai Jiao Tong University. Written informed consent was obtained from all participants. The clinical characteristics of the PCOS and non-PCOS groups are shown in Table 1.

**Table 1:** Biochemical indexes from controls and PCOS patients.

	**Control (*n* = 37)**	**PCOS (*n* = 48)**
Age (year)	27.53 ± 1.32	26.32 ± 0.96
BMI (kg/m^2^)	21.32 ± 0.82	22.29 ± 1.39
Basal FSH (IU/L)	7.19 ± 0.57	6.94 ± 0.42
Basal LH (IU/L)	4.73 ± 0.49	7.54 ± 1.37^*^
Basal E2 (pg/mL)	43.48 ± 5.70	48.54 ± 10.76
Basal T (nmol/mL)	1.08 ± 0.23	1.42 ± 0.22^*^
Basal PRL (μg/L)	13.80 ± 2.07	15.86 ± 2.53
LH/FSH	0.74 ± 0.23	1.66 ± 0.14^*^
AMH (ng/mL)	5.67 ± 0.30	10.58 ± 3.56^**^
AFC	12.38 ± 2.43	29.36 ± 4.64^***^
FBG (nmol/L)	4.84 ± 0.39	5.02 ± 0.15
Number of retrieved oocytes	13.43 ± 1.27	16.33 ± 3.72^*^

E_2_, estradiol; T, testosterone; PRL, prolactin; AMH, Anti-Müllerian Hormone; FBG, fasting blood glucose;

*Data are presented as mean ± SEM*.

* *P < 0.05*,

** P < 0.01 and

*** *P < 0.001 vs. control group*.

### Cell culture

hGCs were collected from the PCOS and non-PCOS subjects after their first IVF/intracytoplasmic sperm injection cycle at our center. At 36 h after triggering, COCs were retrieved via the transvaginal ultrasound-guided aspiration of follicles ≥10 mm. Follicular fluid (FF) from the patients was pooled. The protocol of GC isolation and culture was slightly modified from the previously described method (25). In brief, the FF was immediately centrifuged at 1,000 rpm for 10 mins to avoid post-aspiration cell death. The cell pellet was resuspended with 0.01% phosphate-buffered saline (PBS). The hGCs were purified by density gradient centrifugation with Ficoll-Paque (GE Healthcare Bio-Sciences, UK) and then digested with hyaluronidase (Sigma, USA) at 37°C for 7 mins. The dispersed cells were collected and cultured in Dulbecco's modified Eagle medium /Ham's F12 with 10% fetal bovine serum (Gibco, USA), 100 U/mL penicillin and 100 mg/mL streptomycin sulfate (Invitrogen, USA). The viable cells were seeded at 10^6^ cells per well in a six-well culture plate, at 5 × 10 ^5^ cells per well in a 12-well culture plate or at 1 × 10 ^4^ cells per well in a chamber slide. For hCG stimulation, culture medium was replaced with fresh medium supplemented with 10 IU/mL hCG for 24 h according to a previous study (26). For the signaling inhibition experiment, an ERK inhibitor (PD98059, Selleckchem, USA), AKT inhibitor (LY294002, Selleckchem) or PKA inhibitor (SQ22536 and H89, Selleckchem) was added for 24 h. The culture medium was changed daily for all experiments. Before treatment, hGCs were cultured in serum-free medium for 72 h.

### Immunofluorescence staining

On the third day of culture, cells in a chamber slide (BD Biosciences, USA) were fixed in 4% paraformaldehyde and then permeabilized with 0.4% Triton X-100. After washing, the cells were blocked with normal goat serum (Proteintech, China) for 1 h and then incubated with ATF4 (1:200, RRID: AB_205875, Santa Cruz, USA) overnight at 4°C. After washing with phosphate-buffered saline, Alexa Fluor 594 goat anti-mouse immunoglobulin G (red, 1:200, Proteintech, China) was used as secondary antibodies, and the cells were incubated in the dark for 2 h. The nuclei were counterstained with 4′, 6′-diamidino-2-phenylindole (blue). Staining intensity was examined using a fluorescence microscope.

### Transfection experiment

2 × 10 ^5^ hGCs were transfected with 50 nM siRNA (GenePharma, China) in Opti-MEM (Invitrogen). The nucleotide sequences for ATF4 siRNA were as follows: sense 5′- CUCCCAGAAAGUUUAACAATT−3′; antisense: 5′- UUGUUAAACUUUCUGGGAGTT-3′. The cells were electroporated at 175 V for 5 ms using an NEPA21 electroporator, for transfection. After dilution with Dulbecco's modified Eagle's medium/F12 containing 10% fetal bovine serum and antibiotics, the cells were transferred to a six-well culture plate and were ready for treatment after 72 h of incubation. The knockdown efficiency was determined using quantitative real-time polymerase chain reaction (qRT-PCR) or Western blot assays.

### qRT-PCR

Total RNA was extracted from hGCs using a total RNA isolation kit (FOREGENE, China), the samples were then stored at −80°C for subsequent analysis. RNA concentrations in each sample were determined by calculating the OD260/OD280 ratio using an ultramicro spectrophotometer (Thermo Fisher Scientific, USA). A total of 500 ng of RNA was used for cDNA synthesis with the PrimeScript RT Master Mix Perfect RealTime Kit (TaKaRa, China). Three separate experiments were performed, and each sample was assayed in triplicate. The mean value was used for the determination of mRNA levels by the comparative Ct (2^−ΔΔ*Ct*^) method, with GAPDH as the reference gene. The primer sequences are shown in the Supplemental Table 1.

### Western blot analysis

Total protein was extracted from ovarian tissues, mixed with sodium dodecyl sulfate (SDS) sample buffer, and then boiled for 10 mins. For protein analyses, 30 μg of protein from each sample was loaded onto an SDS polyacrylamide gel for electrophoresis and subsequently transferred onto polyvinylidene fluoride membranes. The membranes were blocked in 5% skim milk for 60 mins at room temperature before incubation overnight at 4°C with the following primary antibodies: anti-ATF4 (1:1,000, Santa Cruz, USA, RRID: AB_2058752), anti-COX2 (1:1,000, Proteintech, China, RRID: AB_2085127), and anti-GAPDH (1:5,000, Abcam, USA, RRID: AB_2107448). The samples were then incubated for 90 mins at room temperature with secondary antibodies (1:5,000, Cell Signaling, USA). Chemiluminescence reagent (Thermo Fisher Scientific, USA) was used to visualize the blots. The representative blots were obtained from three independent experiments.

### Enzyme-linked immunosorbent assay (ELISA) for PGE2

A human PGE2-specific ELISA kit was used in accordance with the manufacturer's protocol (Cayman Chemical, USA). Culture medium was collected, and the PGE2 levels in the culture medium were determined by ELISA kit. The OD values for PGE2 were normalized with the protein concentrations of the corresponding cell lysates. The normalized PGE2 values obtained from the treated cells are relative to those of the control cells. The linear range of the PGE2 concentrations was 7.8–1,000 pg/mL.

### Chromatin immunoprecipitation (ChIP) assay

ChIP assays were performed as described by the manufacturer (Upstate Biotechnology, NY) with some modifications. Briefly, hGCs were cultured with or without 10 IU/ml hCG for 24 h. Chromatin solutions were sonicated, incubated with an anti-ATF4 antibody or control IgG, and rotated overnight at 4°C. Then, the enriched chromatin DNA was purified and subjected to PCR analysis. To amplify the human *COX2* promoter region containing the ATF4-binding site, the following primer sets were used: *COX2* ChIP Forward, 5′-AGCTTCCTGGGTTTCCGATTTTCT-3′ and *COX2* ChIP Reverse, 5′-CCCTGCTGAGGAGTTCCTGGA-3′.

### Animal studies

*Atf4*-RNAi-Lentivirus was injected intrabursally into 8-week-old female Wistar rat ovaries with a 10 μl-syringe (Gaoge, China). The targeting sequence of the shRNA was 5′- GACAGCTAAAGTGAAGACTGA−3′. The recombinant lentivirus of small interference RNA targeting *Atf4* (*Atf4*-RNAi-Lentivirus) and the control lentivirus (GFP-lentivirus) were prepared and titered to 10^9^ TU/mL (transfection unit). The needle was inserted slowly and held in place for 5 min. Each ovary was injected twice at different sites with 10 μl each time. Ten days after lentivirus injection, each rat was injected intrabursally with 40 IU pregnant mare's serum gonadotropin (PMSG; PROSPEC, USA) to stimulate follicle development for 48 h, then, 10 IU hCG was given to induce ovulation as described before (27). The rats were sacrificed by decapitation at 16 h after hCG administration. All experimental procedures were approved by the Institutional Review Board of Ren Ji Hospital, School of Medicine, Shanghai Jiao Tong University. All rats received humane care in accordance with the National Institutes of Health Guide for the Care and Use of Laboratory Animals.

### Statistical analysis

The data are presented as the mean ± SEM. All analyses were conducted using SPSS 21.0 software for Windows (IBM, USA). All data was tested for normality. Paired group means were compared for statistical significance using Student's *t*-tests, while multiple means were compared by analysis of variance (ANOVA) followed by Tukey's *post hoc* tests. Values of *P* < 0.05 were considered statistically significant.

## Results

### The expression of ATF4 in hGCs from women with PCOS was decreased

**Figure 1:**
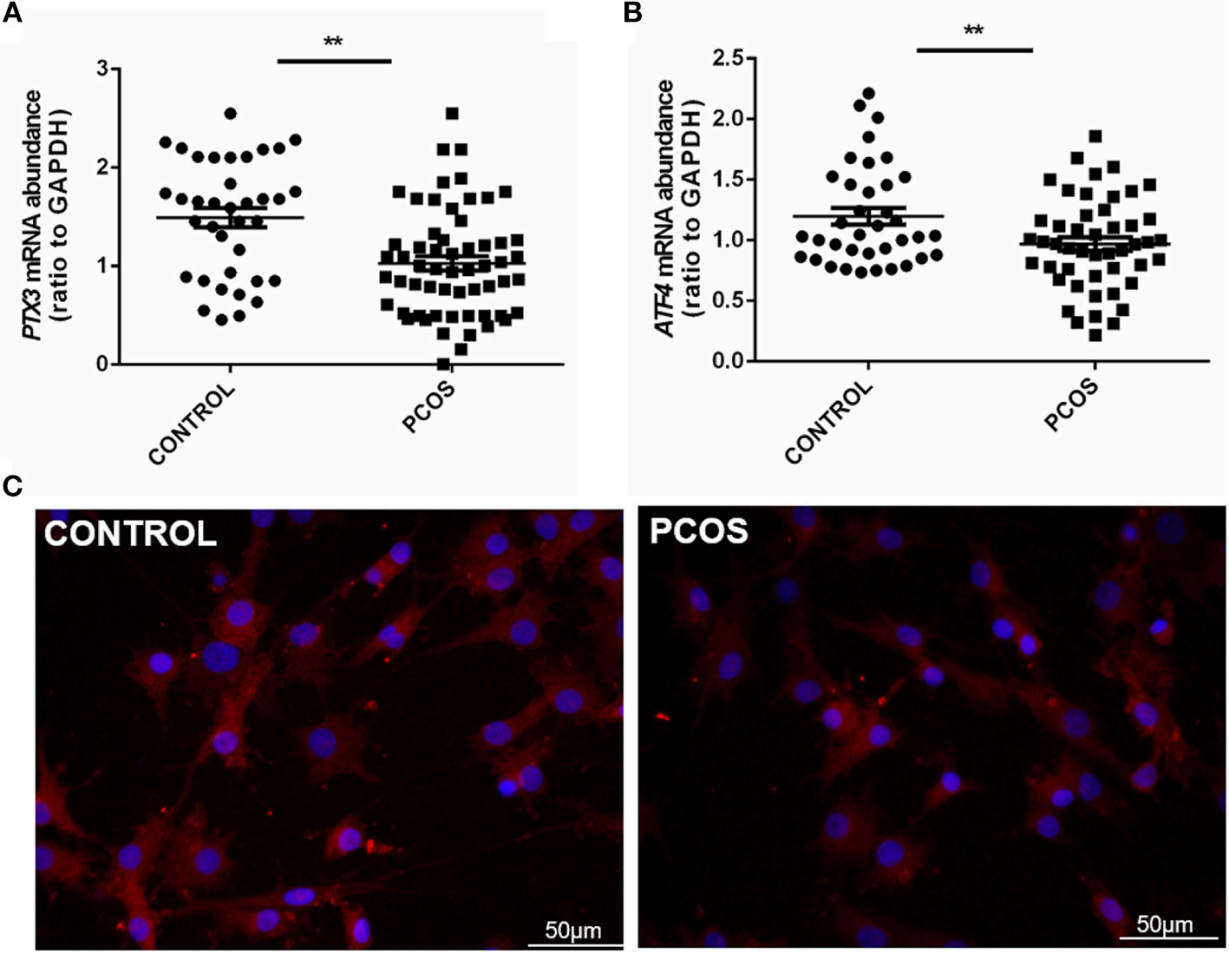
*PTX3* and ATF4 expression in hGCs from women with PCOS was decreased. **(A,B)** Primary hGCs were isolated from healthy controls (*n* = 37) and PCOS patients (*n* = 48). *PTX3* and *ATF4* mRNA levels in primary hGCs were analyzed by qRT-PCR. ^**^P < 0.01 vs. the control group. **(C)** The representative images of immunofluorescence staining of ATF4 were performed in a non-PCOS and a PCOS patient. ATF4 (red) is positively expressed in hGCs. Cellular nuclei (blue) were stained with 40, 6-diamidino-2-phenylindole (DAPI).

We first determined whether *ATF4* expression was changed in clinical PCOS patients. As shown in Table 1, compared with the controls, PCOS patients were characterized by increased testing indexes, including basal LH, LH/FSH, T and AMH levels. qRT-PCR was performed to measure *ATF4* mRNA in primary hGCs. As shown in Figure 1A, pentraxin 3 (*PTX3*) levels were lower in the PCOS group than in the non-PCOS group, in accordance with previous research results (28). The mRNA and protein abundance of ATF4 were lower in the PCOS group than in the control group (Figures 1B,C). Therefore, *in vitro* and *in vivo* experiments were performed to explore the physiological role of ATF4.

### ATF4 regulated a variety of genes associated with ovulatory response in hGCs

**Figure 2:**
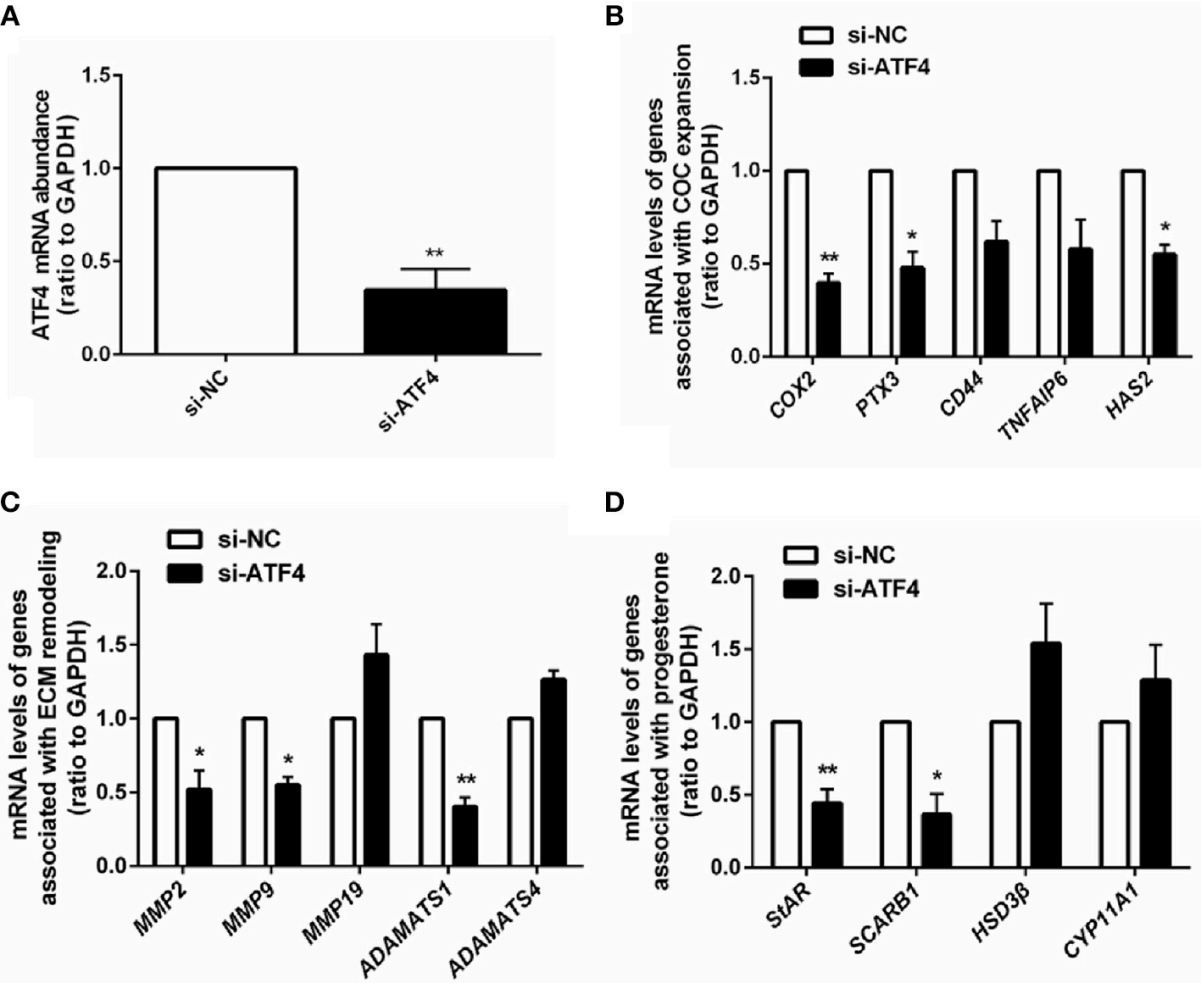
ATF4 in hGCs regulated a variety of genes associated with ovulatory response. **(A)** 2 × 10^5^ hGCs were transfected with 50 nM control siRNA (si-NC) or ATF4 siRNA (si-ATF4) for 48 h. The efficacy of siRNA-mediated *ATF4* knockdown was examined in hGCs. **(B–D)** The expression levels of genes related to COC expansion, ECM remodeling and progesterone synthesis were analyzed by qRT-PCR. ^*^P < 0.05 and ^**^P < 0.01 vs. the si-NC group.

To further illuminate the role of ATF4 in ovulation regulation, siRNA-mediated gene knockdown was used to down-regulate the endogenous expression of *ATF4* in hGCs. The qRT-PCR results showed a significant decrease in *ATF4* expression in hGCs (Figure 2A). More importantly, we found that several ovulation-related genes were significantly altered. The mRNA levels of genes associated with COC expansion in hGCs, including *COX2, PTX3, CD44, TNFAIP6*, and *HAS2*, were decreased when *ATF4* was deficient (Figure 2B). Other genes that play a pivotal role in extracellular matrix (ECM) remodeling were detected through qRT-PCR. The mRNA expression of *MMP2, MMP9*, and *ADAMTS1* in hGCs was significantly impeded when *ATF4* knockdown was achieved with RNAi (Figure 2C). The mRNA expression levels of genes involved in progesterone synthesis, such as *StAR* and *SCARB1*, were also decreased, while the expression levels of *HSD3*β and *CYP11A1* were barely changed (Figure 2D). Altogether, these results indicated that ATF4 participates in regulating the expression of ovulatory genes.

### AKT signaling was required for HCG-induced ATF4 expression in hGCs

**Figure 3:**
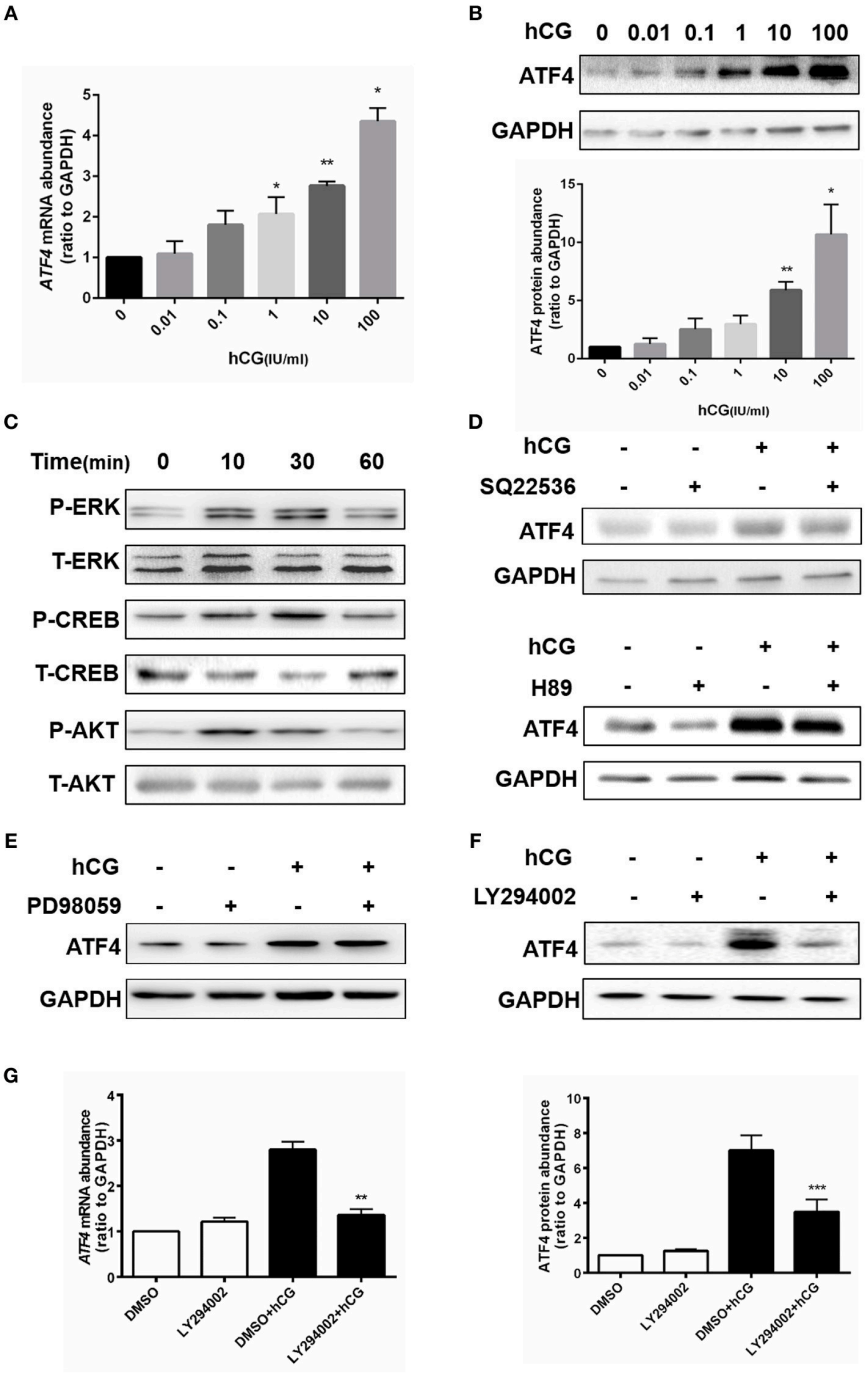
AKT signaling was required for hCG-induced ATF4 expression in hGCs. **(A,B)** Cells were treated with 0, 0.01, 0.1, 1, 10, and 100 IU/mL hCG for 24 h as indicated. ATF4 mRNA and protein expression levels were analyzed in hGCs. ^*^P < 0.05 and ^**^P < 0.01 vs. the 0 IU/mL group. **(C)** Cells were treated with 10 IU/mL hCG for 0, 10, 30, or 60 mins. CREB, ERK1/2, and AKT phosphorylation levels were examined by western blot. **(D,E)** Cells were pre-treated with DMSO (vehicle control), 10 μM SQ22536, 10 μM H89, or 10 μM PD98059 for 1 h and then treated with DMSO or 10 IU/mL hCG for 24 h. ATF4 protein expression levels in hGCs were determined by western blot. **(F,G)** Cells were pre-treated with DMSO or 10 μM LY294002 for 1 h and then treated with DMSO or 10 IU/mL hCG for 24 h. ATF4 expression in hGCs was determined by qRT-PCR and western blot. ^**^P < 0.01 and ^***^P < 0.001 vs. the DMSO + hCG group.

Given that ATF4 plays such a critical role in regulating genes essential for ovulation, the effects of hCG on ATF4 expression in hGCs were then examined. Cultured hGCs were treated with different concentrations of hCG. The results obtained from qRT-PCR and Western blot analyses showed that hCG positively regulated the mRNA and protein abundance of ATF4 in hGCs in a dose-dependent manner. In particular, hCG treatment at concentrations higher than 10 IU/mL significantly induced ATF4 expression in hGCs (Figures 3A,B). These results collectively revealed that ATF4 expression in hGCs was elevated after hCG stimulation. The mechanisms underlying the regulation of ATF4 by hCG remain unclear. Herein, several signaling pathways, including the PKA/CREB, ERK1/2 and PI3K/AKT signaling pathways, that might be involved in this regulation were examined. As shown in Figure 3C, the phosphorylation levels of CREB, ERK1/2, and AKT were increased considerably in hGCs treated with 10 IU/mL hCG for 10 and 30 min. The data suggested that these signaling pathways were activated under hCG stimulation. To further verify the exact pathway involved in activating ATF4 after hCG stimulation, pharmacological inhibitors were used to block the activation of signaling pathways. Inhibiting CREB by SQ22536 or H89 did not affect the protein expression of ATF4 upon hCG treatment (Figure 3D). Treating cells with PD98059, a specific inhibitor for MEK, did not attenuate the induction of ATF4 expression upon hCG exposure (Figure 3E). Notably, the PI3K inhibitor LY294002 significantly impaired hCG-induced increase in ATF4 expression (Figure 3F). The qRT-PCR results were consistent with the Western blot results (Figure 3G). Taken together, these data indicated that PI3K/AKT signaling pathway activation was required for the hCG-induced increase in ATF4 expression in hGCs.

### ATF4 could directly bind to the COX2 promoter, and *ATF4* knockdown attenuated hCG-induced COX2 expression and PGE2 production in hGCs

**Figure 4:**
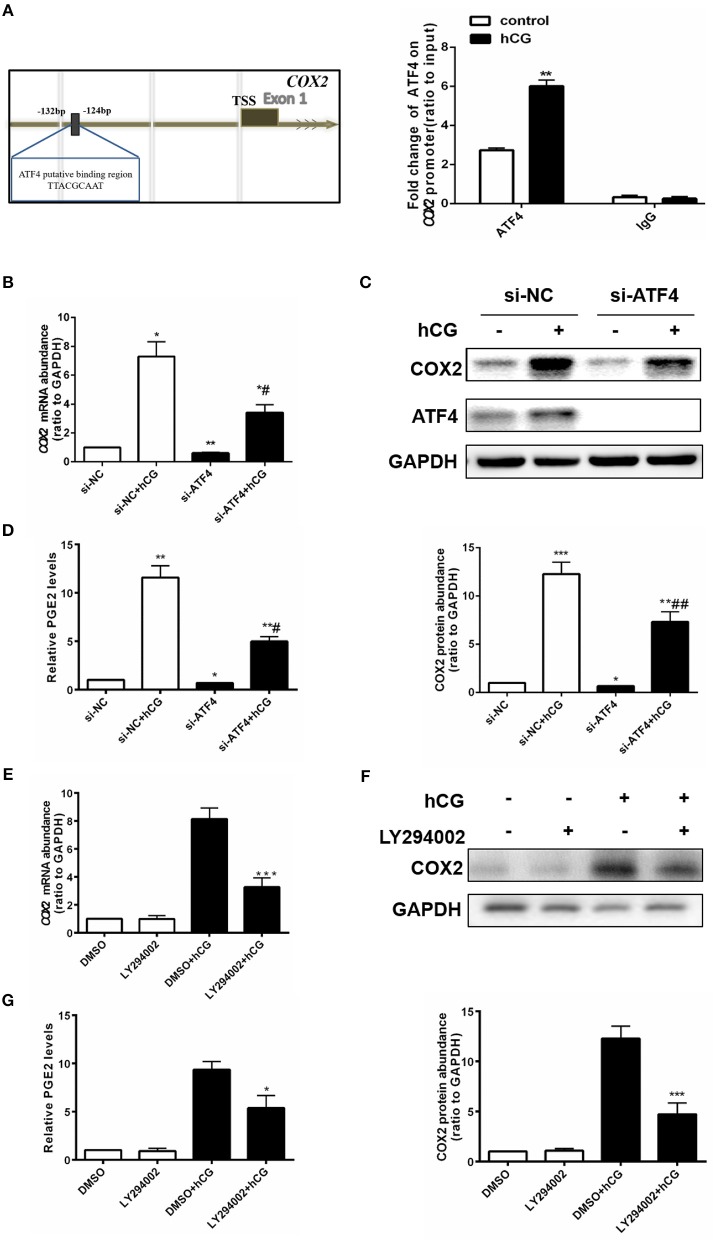
ATF4 could directly bind to the *COX2* promoter, and *ATF4* knockdown attenuated hCG-induced COX2 expression and PGE2 production in hGCs. **(A)** Cells were treated with DMSO or 10 IU/mL hCG for 24 h. Normalized inputs of chromatin DNA from hGCs were pulled down with ATF4 or negative IgG antibodies. DNA templates were amplified by PCR. ^**^P < 0.01 vs. the DMSO group. **(B)** Cells were transfected with 50 nM si-NC or si-ATF4 for 48 h and then treated with DMSO or 10 IU/mL hCG for 24 h. *COX2* mRNA levels were detected by qRT-PCR analysis. **(C)** COX2 and ATF4 protein levels were detected by western blot analysis. **(D)** PGE2 levels in the culture supernatants were measured by ELISA. ^*^P < 0.05, ^**^P < 0.01, and ^***^P < 0.001 vs. the si-NC group, ^#^*P* < 0.05 and ^##^*P* < 0.01 vs. the si-NC + hCG group. **(E)** Cells were pre-treated with DMSO or 10 μM LY294002 for 1 h and then treated with DMSO or 10 IU/mL hCG for 24 h. *COX2* mRNA levels were detected by qRT-PCR analysis. **(F)** COX2 protein levels were detected by western blot analysis. **(G)** PGE2 levels in the culture supernatants were measured by ELISA. ^*^P < 0.05 and ^***^P < 0.001 vs. the DMSO + hCG group.

ATF4 can bind to the *COX2* promoter (23), and COX2-derived PGE2 plays a critical role in regulating ovulation (29). Thus, we further investigated whether ATF4 directly modulated COX2 by binding to its promoter region in hGCs. We identified a putative ATF4-binding site (5′ -TTACGCAAT-3′) between nucleotides −132 and −124 on COX2 promoter sequences, by using TESS for predicting transcription-factor-binding sites in DNA sequences(Figure 4A). hGCs were cultured without or with 10 IU/mL hCG for 24 h. The enriched fragments in the *COX2* promoter utilizing an ATF4 antibody were determined through qRT-PCR. The results demonstrated that the fragments to which ATF4 bound in the *COX2* promoter were significantly increased in hCG-treated hGCs, suggesting that ATF4 up-regulated COX2 expression mainly by enhancing the transcriptional activity of *COX2* via binding to its promoter regions (Figure 4A). As shown in Figures 4B,C, the knockdown of *ATF4* by specific siRNA decreased the basal levels of COX2, which was consistent with the results shown in Figure 2B. hCG effectively stimulated the mRNA and protein expression of COX2, while *ATF4* knockdown dampened the hCG-induced increase in COX2 abundance (Figures 4B,C). PGE2 followed a similar trend as COX2 (Figure 4D). As shown in Figures 4E–G, the results also showed that hCG increased COX2 expression and PGE2 levels depending on PI3K/AKT pathway activation. To sum up, these data indicated that ATF4 directly bound to the *COX2* promoter and that *ATF4* knockdown could attenuate hCG-induced COX2 expression and PGE2 production via the PI3K/AKT signaling pathway.

### Atf4 in rat ovaries regulates ovulation function

**Figure 5:**
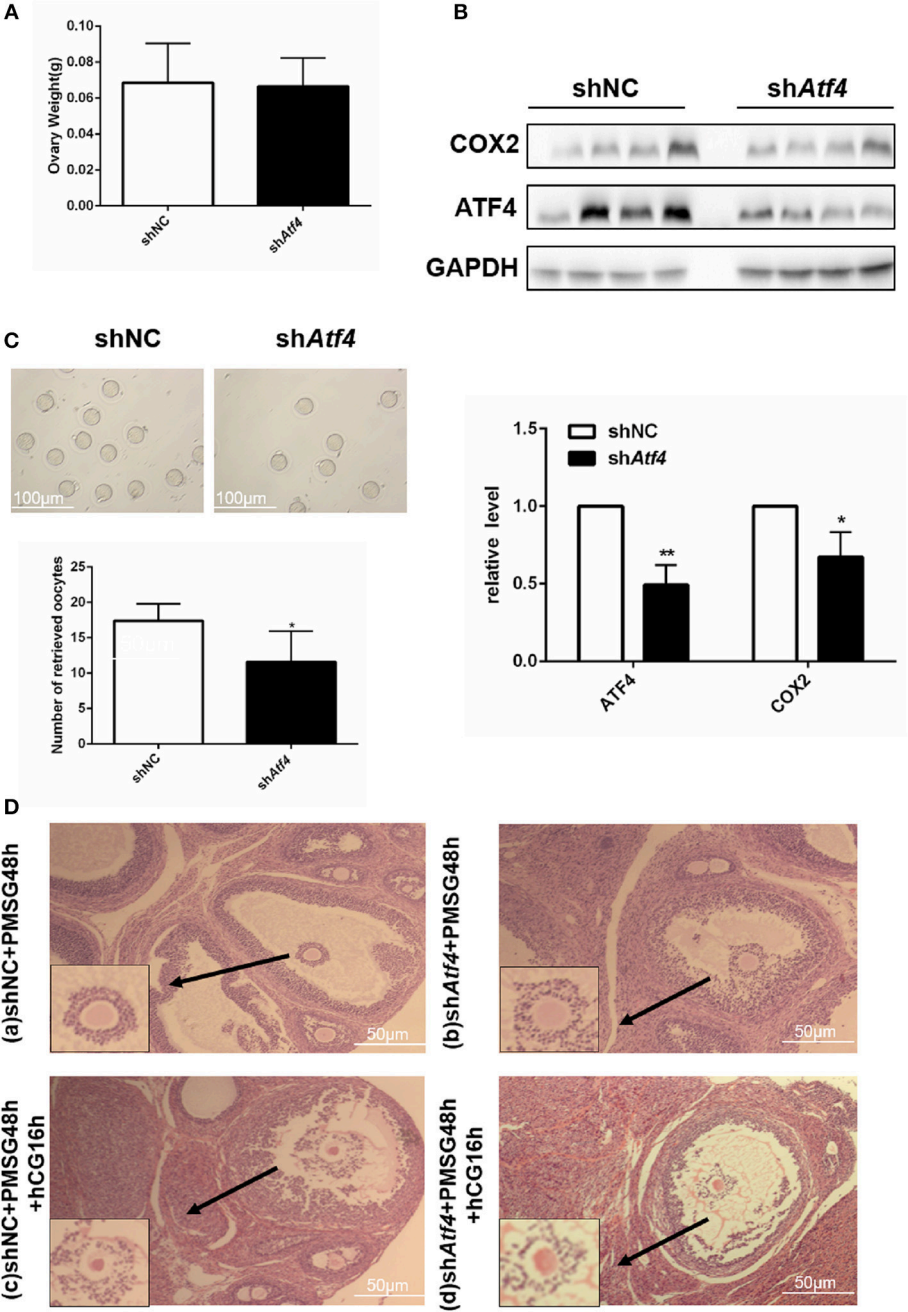
*Atf4* in rat ovaries regulates ovulation function. **(A)** Rats were transfected with lentiviruses encoding sh*Atf4* or shNC by intrabursal injection. After 7 days, the rats were sacrificed. Ovarian weights were measured. **(B)** ATF4 and COX2 protein levels in rat ovaries were quantitated by western blot. ^*^P < 0.05 vs. shNC. **(C)** After transfection, the rats were treated with PMSG for 48 h and then with hCG for 16 h for superovulation. The number of oocytes retrieved from the rat fallopian tubes was measured. ^*^P < 0.05 and ^**^P < 0.01 vs. the shNC group. **(D)** a,b Hematoxylin stained preovulatory follicles 48 h was detected after the PMSG treatment (before hCG injection). **(D)** c,d Hematoxylin stained preovulatory follicles 16 h was detected after induction of ovulation by hCG injection.

To verify the function of *Atf4 in vivo, Atf4*-deficient ovarian tissues were established in rats via the intrabursal injection of lentivirus sh*Atf4*. Lentivirus sh*Atf4* successfully reduced ATF4 expression in rat ovaries without affecting ovary weights (Figures 5A,B). Then, rats were injected with PMSG, followed by hCG injection, 48 h later. After another 16 h, the rats were sacrificed, and the ovarian tissues and fallopian tubes were harvested. *Atf4* knockdown effectively reduced the number of oocytes retrieved from the fallopian tubes (Figure 5C and Table 2). When sh*Atf4* rats were treated with PMSG and hCG, preovulatory follicular development was normal (Figure 5D)a,b. However, COC expansion was impaired slightly (Figure 5D)c,d. Furthermore, when COC expansion was examined *in vitro*, the physiological mediators of COX2 (30), was less effective in inducing expansion of COCs collected from sh*Atf4* compared with shNC (Figure 5B).The phenomenon of oligo-ovulation in *Atf4*-deficient rats could be explained by the incorrect expansion of COC. Altogether, these results indicated that *Atf4* was required for the execution of ovulation in rat ovaries.

**Table 2:** Number of retrieved oocytes from rat fallopian tubes.

	**shNC group (*n* = 4)**	**sh*Atf4* group (*n* = 4)**
Number of retrieved oocytes	17.40 ± 1.077	11.60 ± 1.939^*^

*Data are presented as mean ± SEM*.

* *P < 0.05 vs. shNC group*.

## Discussion

In the present study, we demonstrated that ATF4 was expressed in hGCs; this finding was indispensable for determining the physiological function of ATF4. Whether ATF4 expression was changed in PCOS patients remained unclear. Thus, we further investigated the role of ATF4 in PCOS by analyzing clinical samples. First, we determined that *PTX3* expression was lower in PCOS, and we regarded this as a positive control result, and this finding was consistent with previous reports (28). Consistently, we found that the levels of ATF4 in primary hGCs from PCOS patients were decreased. These data implied that the aberrant expression of ATF4 might be correlated with PCOS.

The normal expansion of COC is required for ovulation, which requires the abundant expression of transcripts, such as COX2, PTX3, CD44, TNFAIP6, and HAS2 (31). Moreover, variations in the ECM secreted from cumulus cells are also essential for oocyte release. Several classes of proteinases have been reported to regulate ovarian ECM remodeling, including MMPs, the plasmin/plasminogen activator system, and ADAMTS (32). ATF4 has been recently identified as an important controller of reproduction (13, 33). The absence of ATF4 in hGCs resulted in a decrease in the expression of multiple genes involved in COC expansion, ECM remodeling, and progesterone production. However, not all markers related to ovulation were downregulated. Ovulation is a highly complex, and primary physiological process that multiple signals involved in. ATF4 could just play important role in this process.

hCG, a hormone secreted by the placenta, can induce progesterone and ovulation production in the ovaries, which is consequentially used for the treatment of infertility (12, 34). Given that ATF4 plays such a critical role in the ovulation process, the effects of hCG on ATF4 expression in hGCs were then tested. Our subsequent results showed that hCG significantly induced ATF4 expression in hGCs in a concentration-dependent manner. The mechanisms underlying the regulation of ATF4 by hCG are not known. In the present work, several signaling pathways that might be involved in this process were examined. We found that hCG-induced ATF4 up-regulation was related to activation of the PI3K/AKT pathway rather than the PKA/CREB and ERK1/2 signaling pathways.

ATF4 can induce COX2 expression in the kidney, emphasizing the potential correlation between ATF4 and COX2 (21, 22), some studies indicate ATF4 induces ovarian granulosa cell damage and inhibits ovarian follicle activation (13, 35). Previously, we identified the key role of ATF4 in modulating the expression of COX2 in hGCs (Figure 2B). Consistently, hCG could also induce COX2 expression (18, 19). The increases in COX2 and PGE2 levels are representative indicators of ovulation (36, 37). Mechanistically, we found that ATF4 deficiency impaired the hCG-mediated induction of COX2 and PGE2 expression and ATF4 likely exerts its effects by directly binding to the promoter of *COX2* in hGCs. ATF4 could form heterodimers with members of C/EBP families, including C/EBPα and C/EBPβ, which are essential for ovulation (9, 10). The putative ATF4 binding region at the COX 2 promoter could be partially overlapping with the C/EBP families, and *more subjects* should *be further required to prove our concluded results*. Thus, these findings suggested that ATF4 is closely involved in the regulation of ovulatory indicators.

An *in vivo* study was also performed to investigate the role of *Atf4* in ovulation. Due to the high efficacy and long-term stability of shRNA, we employed lentivirus sh*Atf4*, which could stably reduce the expression of *Atf4* in rat ovaries. Intrabursal injection is a promising method for ensuring the local knockdown of target genes with high efficacy. Compared to the mock transfection, *Atf4* knockdown considerably reduced the number of oocytes retrieved from rat fallopian tubes. These results collectively support the pivotal role of *Atf4* in maintaining normal ovulation in rats.

**Figure 6:**
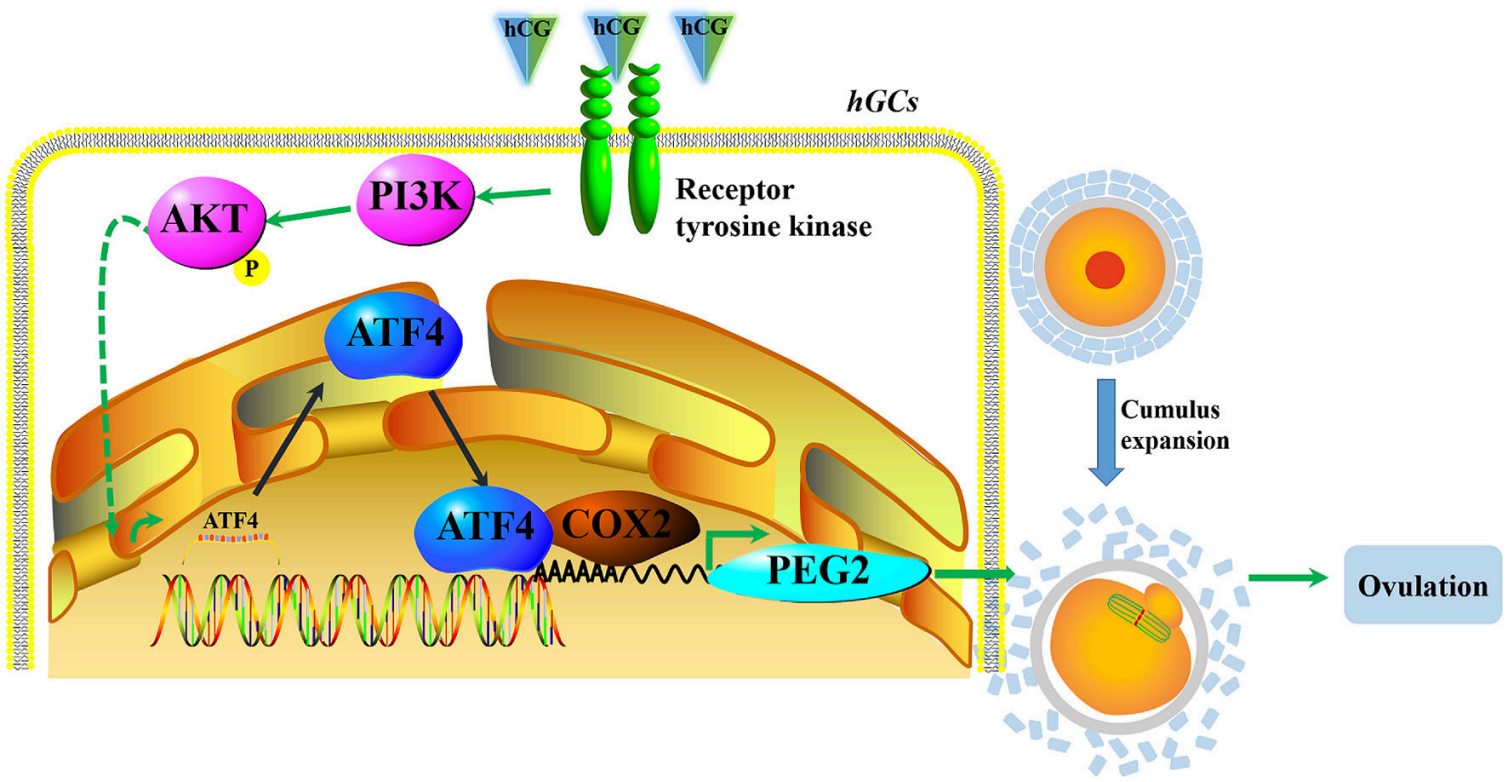
A proposed working model underpinning that decreased ATF4 disrupted ovulation. hCG activated the PI3K/AKT signaling pathway and then stimulated ATF4 expression in hGCs. The increased ATF4 transcripts directly bound to the promoter of *COX2*, which enhanced the transcription of *COX2* and the synthesis of PGE2 to consequently facilitate COC expansion and ovulation.

In conclusion, our results demonstrate that hCG could activate the PI3K/AKT signaling pathway and then stimulate ATF4 expression in hGCs. The increased ATF4 transcripts directly bound to the promoter of *COX2*, which enhanced the transcription of *COX2* and the synthesis of PGE2 to consequently facilitate COC expansion and ovulation (Figure 6). Moreover, decreased ATF4 expression in hGCs is correlated with PCOS. *In vivo*, we confirmed that the knockdown of *Atf4* reduced ovulation and COC expansion which could be related to the ovulation disorder of PCOS. Our study provides a novel insight into the mechanisms underlying the formation and development of PCOS. In this case, ATF4 could likely be used as a potential target for the treatment of PCOS in the future.

## Author contributions

FD and YD designed the research. FD, SL, and YH collected the clinical patients' samples. FD, JL, SL, and GY performed the experiments. FD, JL, WL, and YD analyzed the data. FD, Z-JC, and YD wrote the article.

### Conflict of interest statement

The authors declare that the research was conducted in the absence of any commercial or financial relationships that could be construed as a potential conflict of interest.

## Abbreviations

ATF4: activating transcription factor 4
ChIP: chromatin immunoprecipitation
CL: corpus luteum
COC: cumulus-oocyte complex
COX2: cyclooxygenase-2
DAB: diaminobenzidine
ECM: extracellular matrix
ELISA: enzyme-linked immunosorbent assay
ER: endoplasmic reticulum
FF: follicular fluid
hCG: human chorionic gonadotropin
hGCs: human granulosa cells
IVF-ET: *in vitro* fertilization-embryo transfer
LH: luteinizing hormone
OD260: optical density at 260 nm
PCOS: polycystic ovary syndrome
PGE2: prostaglandin E2
PTX3: pentraxin 3.
