# Supplementary material for: ATF4 Contributes to Ovulation via Regulating COX2/PGE2 Expression: A Potential Role of ATF4 in PCOS
Source: Front Endocrinol (Lausanne). 2018 Nov 15;9:669. doi: 10.3389/fendo.2018.00669 (PMC6249970; doi:10.3389/fendo.2018.00669)
Supplement: Supplementary file 1 [file Table_1.DOCX]

SUPPLEMENTAL TABLE 1 Primer Sequences Used for PCR

| Gene | Species |  | Sequences |
| --- | --- | --- | --- |
| *GAPDH* | Human | Forward | 5’-GCACCGTCAAGGCTGAGAAC-3’ |
|  |  | Reverse | 5’-TGGTGAAGACGCCAGTGGA-3’ |
| *ATF4* | Human | Forward | 5’-ATGACCGAAATGAGCTTCCTG-3’ |
|  |  | Reverse | 5’-GCTGGAGAACCCATGAGGT-3’ |
| *COX2* | Human | Forward | 5’-CTGGCGCTCAGCCATACAG-3’ |
|  |  | Reverse | 5’-CGCACTTATACTGGTCAAATCCC-3’ |
| *PTX3* | Human | Forward | 5’-TCTCTGGTCTGCAGTGTTGG-3’ |
|  |  | Reverse | 5’-TGAAGAGCTTGTCCCATTCC-3’ |
| *CD44* | Human | Forward | 5’-CTGCCGCTTTGCAGGTGTA-3’ |
|  |  | Reverse | 5’-CATTGTGGGCAAGGTGCTATT-3’ |
| *TNFAINP6* | Human | Forward | 5’-TTTCTCTTGCTATGGGAAGACAC-3’ |
|  |  | Reverse | 5’-GAGCTTGTATTTGCCAGACCG-3’ |
| *HAS2* | Human | Forward | 5’-CTCTTTTGGACTGTATGGTGCC-3’ |
|  |  | Reverse | 5’-AGGGTAGGTTAGCCTTTTCACA-3’ |
| *MMP2* | Human | Forward | 5’- GATACCCCTTTGACGGTAAGGA-3’ |
|  |  | Reverse | 5’-CCTTCTCCCAAGGTCCATAGC-3’ |
| *MMP9* | Human | Forward | 5’-AGACCTGGGCAGATTCCAAAC-3’ |
|  |  | Reverse | 5’-CGGCAAGTCTTCCGAGTAGT-3’ |
| *MMP19* | Human | Forward | 5’-GCTTCCTACTCCCCATGACAG-3’ |
|  |  | Reverse | 5’-CCCATATTGTGACAGGTAGTCCA-3’ |
| *ADMATS1* | Human | Forward | 5’- ACTGGAAGCATAAGAAAGAAGCG-3’ |
|  |  | Reverse | 5’- AATTCTGCCATCGACTGGTCT-3’ |
| *ADMATS4* | Human | Forward | 5’-GAGGAGGAGATCGTGTTTCCA-3’ |
|  |  | Reverse | 5’-CCAGCTCTAGTAGCAGCGTC-3’ |
| *StAR* | Human | Forward | 5’-GAGCTCTCTACTCGGTTCTC-3’ |
|  |  | Reverse | 5’-CCATTGTCCTGCTGACTCTC-3’ |
| *SCARB1* | Human | Forward | 5’-CCTATCCCCTTCTATCTCTCCG-3’ |
|  |  | Reverse | 5’-GGATGTTGGGCATGACGATGT-3’ |
| *HSD3β* | Human | Forward | 5’-AGAACGGCCACGAAGAAGAG-3’ |
|  |  | Reverse | 5’-TGGGTCTTAACGCACAAGTGT-3’ |
| *CYP11a1*  *GAPDH* | Human | Forward | 5’-GCAGTGTCTCGGGACTTCG-3’ |
|  | Rat | Reverse  Forward  Reverse | 5’-GGCAAAGCGGAACAGGTCA-3’  5'-AAGATGGTGAAGGTCGGTGT-3'  5'-AGGTCAATGAAGGGGTCGTT-3' |
| *Atf4* | Rat | Forward | 5'-CGATGCTCTGTTTCGAATGG-3' |
|  |  | Reverse | 5'-AGAGGGGCAAAAAGATCACA-3' |
| *Cox2* | Rat | Forward | 5'-GATCACATTTGATTGACAGC-3' |
|  |  | Reverse | 5'-TCCTTATTTCCTTTCACACC-3' |
